# Cold Pressed Oil from Japanese Quince Seeds (*Chaenomeles japonica*): Characterization Using DSC, Spectroscopic, and Monolayer Data

**DOI:** 10.3390/molecules30030477

**Published:** 2025-01-22

**Authors:** Wiktoria Kamińska, Grażyna Neunert, Przemysław Siejak, Krzysztof Polewski, Jolanta Tomaszewska-Gras

**Affiliations:** 1 Department of Physics and Biophysics, Faculty of Food Science and Nutrition, Poznan University of Life Sciences, 60-637 Poznan, Poland; wiktoria.kaminska@up.poznan.pl (W.K.); grazyna.neunert@up.poznan.pl (G.N.); przemyslaw.siejak@up.poznan.pl (P.S.); 2 Department of Food Quality and Safety Management, Faculty of Food Science and Nutrition, Poznan University of Life Sciences, 60-624 Poznan, Poland; jolanta.tomaszewska-gras@up.poznan.pl

**Keywords:** UV–vis, excitation–emission fluorescence, FTIR, DSC, monolayer studies, DSC deconvolution

## Abstract

The cold-pressed oil from Japanese quince seeds (JQSO) is notable for its favorable fatty acid profile, low oxidation rate, and bioactive compounds like antioxidants, sterols, and carotenoids. This study offers a detailed molecular-level physical characterization of JQSO and its minor components using differential scanning calorimetry (DSC), Langmuir monolayer studies, and various spectroscopic methods, including UV–vis absorption, fluorescence, and FTIR. DSC analysis identified five peaks related to triglyceride (TG) fractions and provided insights into the melting and crystallization behavior of JQSO. The Langmuir monolayer studies revealed high compressibility, indicative of superior emulsification properties. Viscoelastic modulus measurements suggested strong intermolecular interactions, contributing to the oil’s resilience under stress—an attribute typical of oils high in saturated or monounsaturated fatty acids. Spectroscopic methods confirmed the presence of phenolic acids, tocopherols, carotenoids, and their derivatives. The total fluorescence spectra highlighted prominent peaks at 290 nm/330 nm and 360 nm/440 nm, while the total synchronous fluorescence spectra revealed key excitation–emission regions (10–50 nm/300 nm and 40–140 nm/360 nm), corroborating the presence of tocopherols, phenols, polyphenols, flavones, and carotenoids. No evidence of chlorophyll was detected. The ATR-FTIR spectra validated the presence of fatty acids and triacylglycerols, emphasizing a high degree of esterification and the dominance of unsaturated fatty acids in oil structures. The methods used provided the opportunity to perform a label-free, fast, and reliable determination of the properties of JQSO. The findings confirmed that crude, cold-pressed JQSO retains its valuable bioactive components, aligning with previous research on its chemical and physical properties.

## 1. Introduction

Japanese quince (*Chaenomeles japonica* (Thumb.) Lindl. Ex Spach) (JQ) is an ornamental plant originating from Asia. In Europe, it is best adopted in the northern part of Europe, including Poland. The fruit is used to produce candied fruits, juices, wines [1], or jams. According to the findings presented by Gornas et al. [2], the oil content extracted from the seeds ranges from 6% to 16%, depending on the extraction method. Cold-pressed oil from Japanese quince (*Chaenomeles japonica*) seeds (JQSO) is gaining recognition for its unique nutritional and functional properties. Extracted without heat or chemical solvents, it preserves the oil’s bioactive compounds. Its fatty acid profile is dominated by three fatty acids: C18:2 (52%), C18:1 (35%), and C16:0 (10%) [2,3,4]. Therefore, JQSO is a rich source of essential fatty acids, particularly linoleic acid (n-6) and oleic acid (n-9). Additionally, JQSO contains significant amounts of α-tocopherol (97% of the total tocopherol isomers), which is one of the highest among many other oils, as well as carotenoids, with a dominant presence of beta-carotene and polyphenolic compounds [3,5]. These compounds exhibit antioxidant and anti-inflammatory effects [3,6,7]. They enhance the oil’s antioxidant properties, neutralizing free radicals and protecting cells from oxidative stress. The oil also exhibits very good oxidative stability, an important factor for shelf life and usability in various applications. JQSO includes phytosterols, where the most present is β-sitosterol (82%), which acts as a stabilizer of phospholipid bilayers in cell membranes.

Five phenols, (−)-epicatechin, (+)-catechin, chlorogenic acid, rutin, and isoquercitrin, were identified in all three cultivars, Rasa, Darius, and Rondo, using the HPLC method [8]. The predominant phenols in all cultivars were flavan-3-ols (catechin and epicatechin), which account for around 94% of the total amount [8]. It was also reported that all cultivars have accumulated high levels of proanthocyanidins [8]. Similar results were demonstrated where 11 phenols were determined with a distribution of procyanidins (57.8%), (−)-epicatechin (33%), and chlorogenic acid (4.4%) [9]. In addition, 24 phenols were identified in the study of five Chaenomeles species, including JQ, and they reported variations in their antioxidant activity and the number of compounds, such as chlorogenic acid, catechin, epicatechin, and procyanidins B1 and B2 [10]. From the 12 detected phenols in seeds, procyanidin B2 and C1 and (−)-epicatechin were predominant [11].

Compared to other cold-pressed oils like flaxseed or sunflower oil, JQSO offers a unique balance of fatty acids and antioxidants, including its highest tocopherol content among many commonly used oils. This makes it a valuable ingredient not only in food [5] but also in cosmetics and pharmaceuticals [3,12].

In the above-presented studies, all chemical characteristics of JQSO, including the fatty acids composition, presence of sterols, total phenolics, presence of tocopherols, and antioxidants, have been thoroughly described. However, in our opinion, there are some other properties that could further characterize this oil based on physical methods, providing deeper insight into the molecular level of the reported parameters. Knowledge of the physical properties of oil is crucial for several reasons, particularly because these properties directly impact the quality, usability, safety, and nutritional value of the oil. Physical methods provide detailed insights into the physical, chemical, and molecular properties of oils, allowing an evaluation of their purity, phase transitions, thermal stability, and overall quality. These methods are non-destructive and faster than traditional chemical evaluation methods. They are highly sensitive, capable of detecting subtle chemical or physical changes, and offer valuable information on composition, oxidation, and degradation. Additionally, they require minimal sample preparation and do not involve complex or chemical treatments. While chemical methods remain essential for detailed chemical analysis, physical methods serve as a complementary approach due to their speed, precision, and ability to provide comprehensive insights into the oil’s characteristics.

To address these possibilities, we applied differential scanning calorimetry (DSC), Langmuir monolayer studies, and spectroscopic methods, including UV–vis absorption, excitation–emission matrix fluorescence, synchronous fluorescence, and FTIR spectra.

## 2. Results and Discussion

### 2.1. Differential Scanning Calorimetry (DSC) Measurements

Differential scanning calorimetry (DSC) is a widely used calorimetric technique for the characterization of the thermal behavior of oils and fats. The parameters obtained from curves are very often statistically correlated with the composition, chemical parameters, or oxidative stability of edible oils. The DSC technique used on oils and fats allows for a determination of solid fat content, study of polymorphic crystal forms, and obtain enthalpy and thermal properties of the transitions. The information derived from the heat capacity function allows the determination of the enthalpy of the system ΔH, entropy ΔS, and melting temperature T_m_, all of them giving the opportunity to calculate standard Gibbs energy ΔG_0_ for the transition.

**Figure 1:**
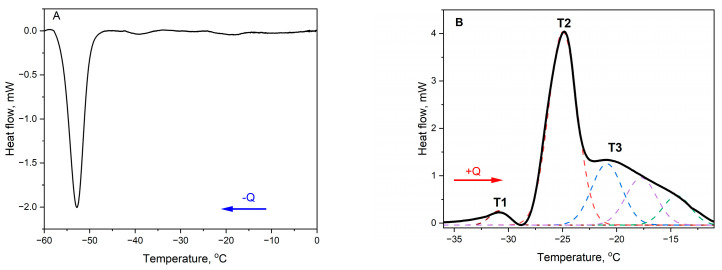
DSC traces of JQSO obtained during (**A**) cooling at 2 °C/min and (**B**) heating at 5 °C/min. In the heating plot, a thick line represents the original recorded signal, whereas dashed lines are the results of the deconvolution procedure to Gaussian peaks.

The cooling and heating profiles of Japanese quince seed oil (JQSO) are shown in Figure 1. It shows a cooling trace (Figure 1A) of one strong and wide peak, with the maximum being at −52.9 °C, indicating the crystallization of different triglycerides (TG) fractions present in the oil. Such a low crystallization temperature indicates the dominant presence of TGs composed of unsaturated fatty acids. However, on the cooling curve, we may also notice barely seen bands at −39 °C and −18 °C, indicating, very probably, the presence of TGs or other structural forms as diglycerides composed of mixed saturated and unsaturated fatty acids.

The melting phase transitions were observed in the form of one very weak peak at −30.9 °C and one sharp peak with a maximum at −28.4 °C overlapped with a lower intensity but a wide band showing a maximum at −21.6 °C, covering the range from −22 °C to −10 °C (Figure 1B). The parameters of the peaks, including the position of maximum temperature T and its enthalpy ΔH, calculated from the crystallization and melting curves, are presented in Table 1. It shows the complex presence of TG structures.

#### Deconvolution of DSC Transitions

To retrieve all thermodynamic components involved in the melting process, we applied the deconvolution method to the entire heating trace. The graphical results after the deconvolution are presented as peaks with colored dashed lines in Figure 1B. From this fitting procedure, we obtained five peaks located at −30.9 °C, −24.9 °C, −20.8 °C, −17.7 °C, and −14.4 °C. Interestingly, their widths around 3 °C, of all fitted peaks are indicating similarity between the structures.

In the DSC method, the area under the curve is proportional to the enthalpy of the transition. Therefore, we may assume that fresh, cold-pressed JQSO contains at least five different TG fractions. Additionally, the area of the last three deconvolved peaks decreases with the rising temperature. This pattern clearly illustrates the sequence of melting intermediates, well-describing the whole melting process of JQSO.

According to the results presented in the literature [6,11], the fatty acids profile of JQSO is dominated by the C18:2, C18:1, and C16:0 fatty acids, which constitute over 90% of the total fats. Therefore, the diversity of formed TGs with a similarity of thermal properties will be rather small. A similar approach to the presence of TGs in olive oil was reported [13].

### 2.2. ATR-FTIR Spectra

**Figure 2:**
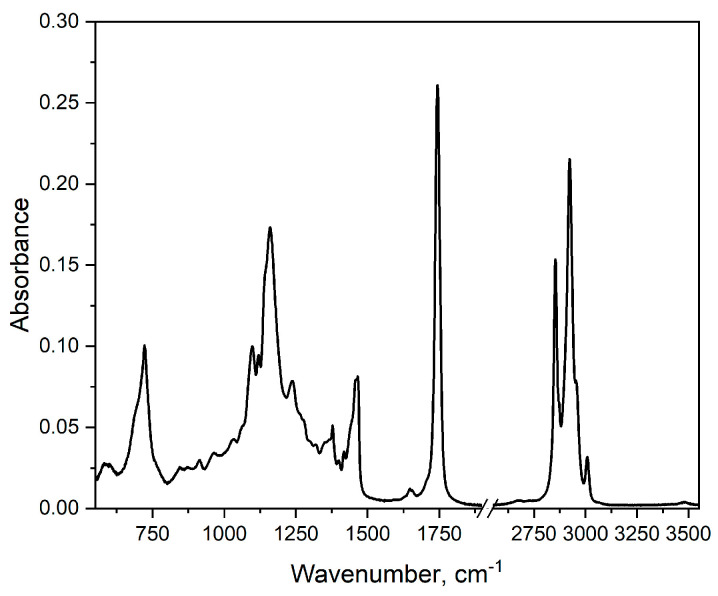
ATR-FTIR spectra of cold-pressed oil from quince seeds.

The measured FTIR spectrum, Figure 2, indicates the presence of groups characteristic for fatty acids and triacylglycerols, i.e., the groups -CH_3_, -CH_2_, -C=O, -OH, and the cis and trans structures of the carbon double bonds (C=C). We may identify a few bands that characterize edible oils. The band in the range 3005–2850 cm^−1^ reflects starching vibrations of C-H in aliphatic groups (CH_2_ and CH_3_). The band around 1740 cm^−1^ is related to the stretching vibration C=O in esters, the main component of TGA. The band in the range 1465–1370 cm^−1^ shows bending vibrations of C-H in CH_2_ and CH_3_. The band in the range 1235–1160 cm^−1^ shows stretching vibrations of C-O in esters. The band around 722 cm^−1^ demonstrates bending vibrations in CH_2_. The analysis of the positions of the characteristic spectral bands makes it possible to evaluate the composition of the obtained oil, especially in terms of the ratio of saturated to unsaturated fatty acids. A good measure of the presence of unsaturated fatty acids was the calculation of the peak ratio of 3008.5/2923 cm^−1^, which expresses the presence of C-H bonds involved in the *cis* C=C bond. A calculated value of 0.148 indicates a relatively low amount of unsaturated fatty as compared to rapeseed 0.18 [14] or elderberry 0.24 [15] oils. The exact position of a weak band between 3006 and 3012 cm^−1^, ascribed to (=C-H) stretching vibrations, depends on the fatty acids’ composition in oil. In this case, the position of 3008.5 cm^−1^ indicates the presence of acyl groups of oleic and linoleic acids [16], and this finding is supported by fatty acids composition studies, where a negligible amount of 18:3 linoleic acid was shown, as reported in the literature [2].

Strong intensities of the bands at 1744, 1235, 1160, and 1120 cm^−1^ indicate the dominant presence of TG in this oil. Additionally, the clearly visible presence of a band at 1120 cm^−1^, which characterizes ester linkage within TG, in this case, is indicative of a high degree of esterification of this oil.

### 2.3. Monolayer Studies

#### 2.3.1. Monolayer Compression

**Figure 3:**
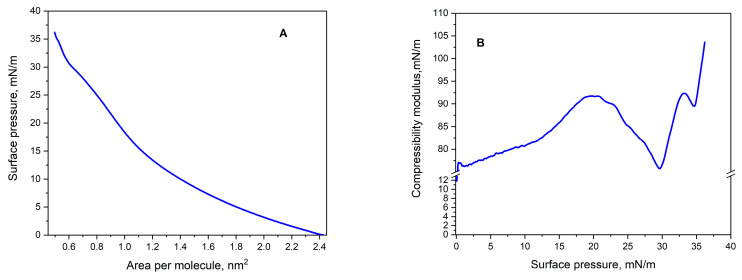
Compression π-A isotherms of Langmuir monolayers (**A**) and compressibility modulus of the surface pressure dependences (**B**) for JQSO.

In this study, the thermodynamic properties of Langmuir monolayers formed by JQSO were measured using surface pressure (π) isotherms as a function of the mean molecular area (Figure 3A). To quantitatively analyze and visualize molecular interactions, the characteristic parameters of the π-A isotherms were determined, including the extrapolated average molecular area (A_EXP_), average molecular area at the collapse point (A_C_), and surface pressure at the collapse point (π_C_). A_EXP_ was determined by extrapolating the tangent to the first linear segment of the isotherm to the point π = 0. The values of the parameters determined based on the recorded isotherm are presented in Table 2.

During monolayer compression, depending on the molecular interactions, various thermodynamic phases may occur, such as expanded liquid (LE), liquid (L), condensed liquid (LC), and solid (S) states [17]. The A_EXP_ value of approximately 1.49 nm^2^ indicates that the molecules of JQSO occupy a relatively large surface area during the initial stages of monolayer formation. This value suggests that the oil molecules are dispersed, with a more loosely arranged spatial distribution compared to other plant oils containing higher amounts of polyunsaturated fatty acids [18,19]. The high A_EXP_ value for this oil may also indicate the presence of long carbon chains in the molecules, which could result in lower intermolecular interactions during the early stages of monolayer formation. The A_C_ value of approximately 0.50 nm^2^ suggests a tendency for the oil molecules to arrange more densely as the surface pressure increases. The reduction in available molecular surface area due to compression implies that the molecules of JQSO tend to form a more compact and ordered structure as the pressure rises, transitioning to a more condensed phase. Meanwhile, the π_C_ value, reaching approximately 36.2 mN/m, suggests that the JQSO monolayer exhibits good mechanical stability. This indicates that the oil molecules effectively interact with each other, forming a robust layer capable of withstanding relatively high surface pressure. Such a result points to the effectiveness of intermolecular interactions within this monolayer, which may be attributed to the structural properties of the oil, including the presence of both saturated and unsaturated fatty acids that facilitate the formation of stable molecular structures. The presence of such compositions of saturated palmitic acid 10% and unsaturated fatty acids, such as oleic 34% and linoleic 52%, was confirmed in the other publications [3,4,8].

#### 2.3.2. The Compressibility Modulus (Cs^−1^)

Using the recorded π-A isotherms and measurements of monolayer behavior under dynamic conditions, the compressibility modulus (C_s_^−1^) and surface viscoelasticity (ε) were calculated by applying Equations (1) and (2), respectively. This analysis provided valuable insights into the stability mechanisms of lipid monolayers as well as their physicochemical characteristics. The relationship between the compressibility modulus C_s_^−1^ and surface pressure π, illustrated in Figure 3B, enabled a comprehensive assessment of the phase states in compressed Langmuir monolayers. The resulting values of C_s_^−1^ and ε are summarized in Table 2.

The C_s_^−1^ modulus represents the monolayer’s response to compression and provides information on its elasticity and the packing density of oil molecules at the air–water interface. Higher Cs^−1^ values indicate a more rigid and stable monolayer, reflecting a tightly packed and condensed oil structure. The classification of the phase states of Langmuir films was performed using the criterion proposed by Davies and Rideal [20]. According to this classification, Cs^−1^ values in the range of 12.5–50 mN∙m^−1^ correspond to the LE phase, 50–100 mN∙m^−1^ define the L phase, 100–250 mN∙m^−1^ indicate the LC phase, and values exceeding 250 mN∙m^−1^ correspond to the S phase. Minima observed in the C_s_^−1^-π plots represent surface pressures at which phase transitions or significant molecular reorganizations take place.

The dependence of Cs^−1^ on π, Figure 3B, reveals the maxima corresponding to the LE and LC phases. The value of Cs^−1^, approximately 103.6 ± 1.9 mN/m, indicates the high rigidity of the JQSO monolayer. The high value of this parameter suggests that the oil molecules tend to form a stable, compact structure under compression, particularly in the LC phase. This means that the oil exhibits stronger intermolecular interactions, resulting in a greater resistance to compression and a more stable monolayer compared to other oils with more compressible structures.

#### 2.3.3. Surface Viscoelasticity Modulus (ε)

In the method of oscillating barriers, the barriers are moved in an oscillatory manner with defined frequency and amplitude. Dilatational interfacial rheology was analyzed under various types of surface deformations, including transient and harmonic changes.

Surface elasticity measurements allow the recorded ε values to be categorized into three distinct zones. In the first zone, where the dynamic elasticity remains below 50 mN/m, long-range forces facilitate the development of a regular structure, with particle distances exceeding their diameters. As the oscillatory motion of the barriers continues, these forces are surpassed, leading to a closer arrangement of particles. In this intermediate zone, the ε values range from 50 to 250 mN/m. The third zone, associated with ε values exceeding 250 mN/m, is characterized by significant compression and a tightly packed monolayer [21].

**Figure 4:**
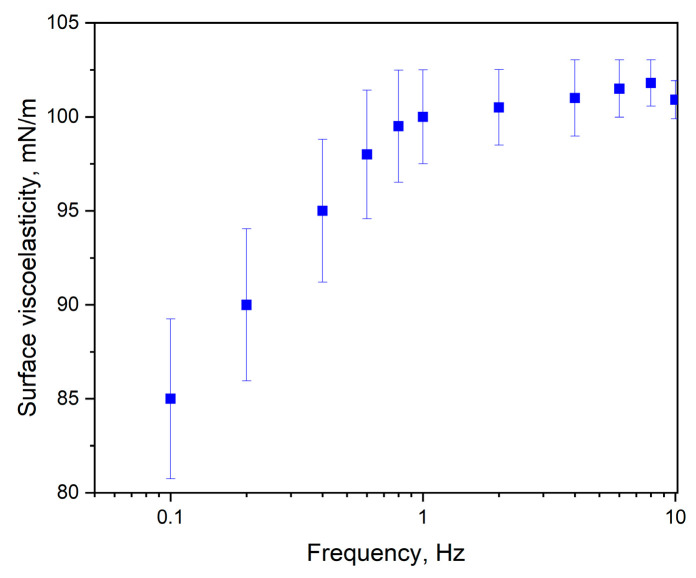
Relation between surface elasticity ε and frequency of deformation [Hz].

The value of ε~100.9 mN/m indicates that the monolayer of JQSO exhibits high resistance to mechanical deformations, combining both elastic and viscous properties. This is shown in Figure 4, where, above 1 Hz of deformation frequency, the monolayer is stable and rigid. Such an ε value suggests that the oil’s layer is not only elastic and capable of recovering its original structure after deformation, but it is also able to resist damage and tearing. Higher ε values are indicative of stronger molecular interactions, which enhance the stability and resistance to stretching of the monolayer. The ε modulus reflects the viscoelastic behavior of the monolayer, encompassing both its elastic (storage) and viscous (loss) components. It provides valuable information about the oil’s capacity to resist mechanical deformation. Elevated ε values signify stronger intermolecular forces, indicating that the oil monolayer is more robust and capable of preserving its structural integrity under stress. This is typically associated with oils containing a high proportion of saturated or monounsaturated fatty acids. In conclusion we may say that JQSO demonstrates favorable physicochemical properties, making it a potentially valuable material for applications requiring stable surface layers.

### 2.4. Absorption and Fluorescence Spectra

#### 2.4.1. Absorption Spectra

Absorption spectra in the UV and visible (UV–vis) regions, with their positions and intensities, provide essential information about the electronic structure of molecules. By measuring the amount of light absorbed at different wavelengths, we obtain the spectra characteristic for each molecule, thus allowing for its identification and determining its concentration, especially in mixtures. In fresh cold-pressed JQSO, the absorption maxima are ascribed to the main groups consisting of tocopherols (270–290 nm), phenolic compounds (250–380 nm), carotenoids (420–520 nm), and flavones (430–450 nm).

**Figure 5:**
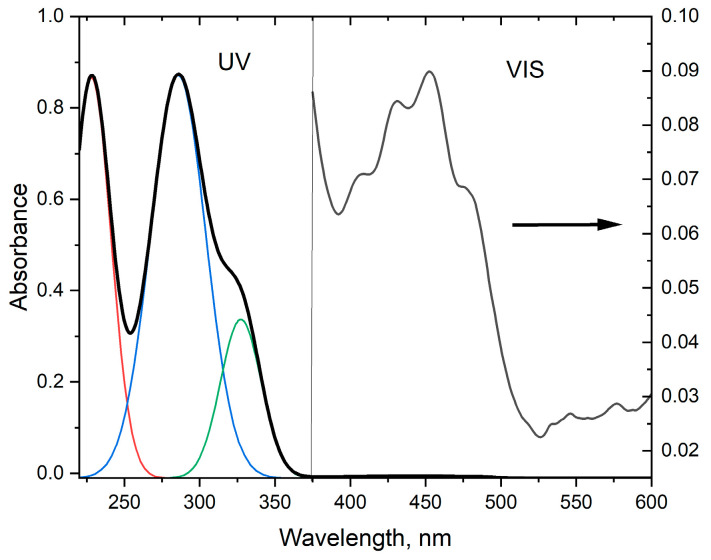
UV–vis absorption spectra of JQSO, taken for 0.2 mm and 0.7 mm layers, for UV and visible spectra, respectively. The intensity of absorbance of the visible spectrum, right axis, is 10 times magnified for better visualization. Red, blue, and green lines in the UV spectrum are the results of the deconvolution procedure to Gaussian peaks.

The absorption spectrum of JQSO in the UV–vis region is shown in Figure 5. It reveals strong maxima in the UV range at 228, 285, and 327 nm, and in the visible region, a wide, very low-intensity band with four peaks at 405, 430, 453, and 480 nm. Despite their low intensity, they indicate the presence of molecules with antioxidant and healthy properties. The strong peak at 228 nm indicates the presence of molecules bearing carbonyl n-π transitions; the peak at 285 nm indicates the presence of tocopherols; and the strong peak at 327 is ascribed to the presence of phenolic compounds. Interestingly, in this oil, no chlorophyll or its derivatives were detected. A similar finding was reported for coconut oil, palm oil, and cottonseed oil. Absorption peaks in the visible region are related to polyphenols, flavonoids, and carotenoids, where the presence of such molecules in JQSO was reported [8,9]. More discussion regarding the characterization of molecules present in JQSO is given in Section 2.4.2.

#### 2.4.2. Fluorescence Spectra

##### Total Luminescence Spectra

**Figure 6:**
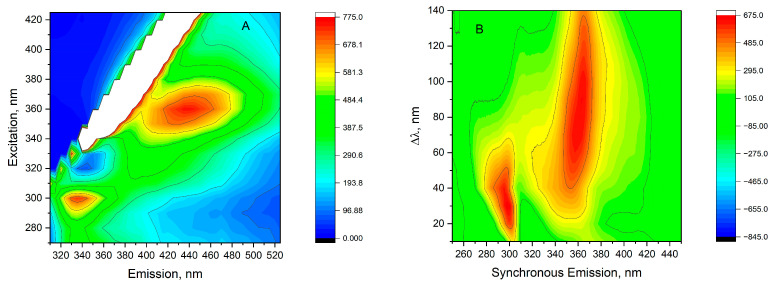
(**A**) Total luminescence spectra; (**B**) total synchronous fluorescence spectra of JQSO recorded in front-face mode.

Figure 6A shows the total luminescence spectrum, which gives rise to the possibility of examining the fluorescent landscape of all fluorescent molecules present in oil. The results are presented as a two-dimensional contour map after removing Rayleigh and Raman scattering, where excitation and emission wavelengths are coordinates. It shows two areas with intense emissions. Such results indicate the presence of a number of fluorescent compounds in the oil sample, and the range of excitation spectra correlates with the absorption spectrum. However, due to the number of fluorescent compounds and the effective overlapping of spectra, it was impossible to reliably identify fluorescent molecules. One area was determined by the observed excitation–emission maxima 300/330 nm, and another with a much bigger area of 360/440 nm. However, the region of intense excitation includes between 340 nm to 370 nm, and emission ranges from 400 nm to 480 nm. In the wavelength range of 360/440 nm, there are included various fluorescent molecules, such as phenols, polyphenols, flavonoids, carotenes, and riboflavin [22]. Phenolic compounds exhibit a peak excitation between 265 and 335 nm, with the peak emission maxima ranging from 358 and 426 nm [23]. Within this wavelength spectrum, emissions from phenolic acids like vanillic, syringic, gallic, *p*-coumaric, *o*-coumaric, cinnamic, and caffeic acids may be detected [3,7,9,11].

The region of 300/336 nm corresponds to the fluorescence of phenolic compounds, which are excited within their absorption bands of 260 to 320 nm. The predominant contributors to observed emissions in this range are tocopherols, absorbing at 280 to 290 nm and emitting at 310 to 330 nm. Additionally, emissions from simple phenolic acids, such as vanillic acid, *p*-coumaric acid, and ferulic acid, as well as polyphenols like catechin and epicatechin, may be identified. These compounds were detected in studies on JQSO, as reported by [3,7,9,11].

The broad fluorescence landscape observed in the studied JQSO highlights the presence of tocopherols, phenols, and flavonoids, many of which are natural antioxidants and/or possess anti-inflammatory properties [24,25]. The total fluorescence spectrum provides a comprehensive overview of the fluorescent components present and serves as a fingerprint for this oil. Each high-intensity fluorescence area is associated with distinct emitting molecules, though spectral overlap complicates the identification of specific fluorophores.

##### Total Fluorescence Synchronous Spectra

To increase the spectral resolution and obtain better discriminant ability in multicomponent samples, the total synchronous fluorescence (TSF) method was applied. In this method, both monochromators were scanned simultaneously with a fixed difference between excitation and emission wavelengths. This gives very characteristic spectra, which usually serve as a fingerprint for a specific molecule. Figure 6B shows the TSF spectra of JQSO in the form of a contour map in coordinates of abscissa as an emission wavelength and Δλ on the ordinate axis. In the TSF contour shown in Figure 6B, we observe two separate areas. The first one, for Δλ, from 10 to 50 nm, excites molecules to emit in the range of 300 nm. The other region with Δλ, from 40 nm to 140 nm, shows a maximum emission in the range of 360 nm, indicating a number of molecules that are excited. This method does not allow for the assignment of specific compounds; however, it clearly indicates a visible excitation range that may excite specific types of molecules fluorescing under such conditions. Such big differences are observed in the Stokes shift, i.e., the difference between excitation and emission maxima, which indicates many interactions existing between fluorescent molecules as well as between fatty acids and these molecules in such a mixture. Furthermore, specific templates obtained from the TSF analysis may be used as a fingerprint or as a tool in oil adulteration studies [26].

## 3. Materials and Methods

### 3.1. Materials

Japanese quince seeds (*Chaenomeles japonica*) of the “Pink Lady” cultivar were collected from the ripped fruits of wild Japanese quince trees in the Kujawy region in Poland. The seeds were dried at 40 °C and then cold-pressed using a screw-pressing machine (Counter Intelligence Oilpresso, Amstelveen, The Netherlands) at a temperature of 45 °C. To remove impurities, the oil samples were centrifuged (MPW Med. Instruments, Warsaw Poland) at 5000 rpm (978× *g*) for 5 min. Before storing, the oil was purged with nitrogen gas (99.99% purity). The oil samples in brown bottles were stored in a freezer at −80 °C until analysis.

### 3.2. Methods

#### 3.2.1. Crystallization and Melting Phase Transition Determination by DSC

The crystallization and melting phase transition of the cold-pressed Japanese quince oil were determined using a PerkinElmer differential scanning calorimeter DSC 7 (Waltham, MA, USA) with an Intracooler II and Pyris software version 11. The detailed procedure of calibration was given in [14].

Oil samples (6–7 mg) were precisely dispensed into aluminum pans (PerkinElmer, No. 0219-0062, Waltham, MA, USA) and hermetically sealed. A sealed, empty aluminum pan served as the reference. The samples underwent an initial cooling process at a scanning rate of 2 °C/min, from 30 °C down to −67 °C, followed by heating at 5 °C/min and back to 30 °C. The onset temperature (Ton), peak temperature (T), and enthalpy (∆H) of the crystallization and melting phase transitions were determined from the cooling and heating curves. The measurements were conducted in duplicate

#### 3.2.2. Absorption and Fluorescence Spectra

The UV–vis absorption spectra of a drop of the sample in a spectral range from 200 to 800 nm were measured using a BioSpec-nano spectrometer (Shimadzu Corp., Kyoto, Japan). The measurements were repeated three times and carried out at two different optical lengths: 0.2 mm and 0.7 mm.

Two-dimensional total luminescence spectra (TFS) were recorded using a front-face sample holder on a Hitachi F-7100 fluorescence spectrometer (Hitachi High-Tech, Delhi, India) at room temperature. Excitation–emission spectra (EES) were generated by scanning the emission monochromator from 210 to 800 nm at 1.0 nm intervals, while the excitation monochromator was scanned from 200 to 750 nm in 10 nm steps.

Total synchronous fluorescence spectra (TSF) were acquired by simultaneously scanning both monochromators at fixed wavelength intervals (Δλ) between the emission and excitation monochromators. The Δλ values ranged from 10 to 140 nm in 10 nm increments. Emission spectra were collected from 210 to 750 nm, and excitation spectra were obtained from 200 to 600 nm.

#### 3.2.3. ATR-FTIR Measurements

FTIR spectra were obtained using a Spectrum Two FTIR spectrophotometer (Perkin Elmer, Waltham, MA, USA) equipped with a Universal ATR (attenuated total reflectance) unit featuring a diamond crystal. Measurements were conducted in the spectral range from 4000 to 500 cm^−1^ with a resolution of 4 cm^−1^ at room temperature. To ensure accuracy, eight replicates were collected for each spectrum.

#### 3.2.4. Monolayer Studies

##### Langmuir Monolayer Method

The investigation of the Langmuir monolayer formed by JQSO was carried out using a computer-controlled Langmuir trough system (KN 2002, KSV NIMA, Helsinki, Finland). The experimental setup consisted of a Teflon trough with an area of 273 cm^2^, equipped with movable hydrophilic barriers made of polyacetyl and a platinum Wilhelmy plate. Ultrapure deionized water with a resistivity of 18.2 MΩ·cm was used as the subphase. A precise volume of JQSO solution in chloroform (purity 99.8%, POCH, Lublin, Poland) was applied onto the surface of the subphase using a Hamilton microsyringe (Hamilton Company, Reno, NV, USA). The compression process was initiated once the chloroform had evaporated. The film was symmetrically compressed at a constant rate of 5 mm/min by the movable barriers. During the experiment, the surface pressure (π) was measured as a function of the molecular area (π-A isotherm). The dependence between the compression modulus (C_S_^−1^) and surface pressure was calculated from the π-A isotherms based on the following equation [27]:

(1) $$C_{s}^{-1}=-A{\left[\frac{\partial \pi }{\partial A}\right]}_{T}$$

where: A is the area per individual molecule, and π is the surface pressure.

##### Oscillatory Dilatational Rheology

The oscillating barrier method was used to investigate the dilatational viscoelasticity of the Langmuir monolayers. This approach involved measuring the surface pressure response to the sinusoidal changes in the surface area with a small amplitude. Initially, the monolayers were compressed to a surface pressure of πc and allowed to equilibrate for 20 min. Following equilibration, the barriers began oscillating, causing minor (1%) variations in the area available for the Langmuir monolayers. The experiments were performed across a frequency range of 0.1 to 1 mHz, with a minimum of 10 oscillation cycles recorded for each frequency. To ensure consistency, a 60 s pause was introduced between consecutive oscillation cycles. The relationship governing dilatational viscoelasticity ε is described by the equation [28,29]:

(2) $$\varepsilon =-A\left[\frac{\partial \pi }{\partial A}\right]$$

#### 3.2.5. Statistical and Graphical Programs

The graphs, calculations, and chemometric analysis were carried out using OriginPro 2024b, ver.10.1.5 (OriginLab Corporation, Northampton, MA, USA) software.

## 4. Conclusions

The results of the applied molecular physics characteristics to already well-known chemical properties of crude cold-pressed oil from Japanese quince seeds add more information on the properties unavailable from previous publications. The DSC, monolayer, and spectroscopic methods provided the opportunity to carry out a label-free, fast, and reliable determination of cold-pressed JQSO properties. The DSC study provided the thermodynamic parameters of the observed transition during cooling and heating, allowing a determination of the temperatures of crystallization or the melting temperature of triglycerides (TG) formed in oil. The data revealed that TG is dominantly composed of unsaturated fatty acids. Deconvolution of the DSC trace obtained during heating identified the presence of five primary TG structures. Fluorescence studies using the total luminescence and total synchronous fluorescence methods provided a comprehensive profile of the minor compounds present in JQSO. These techniques confirmed and visualized the presence of numerous bioactive compounds, indicating high concentrations of tocopherols, phenolics, flavones, and carotenes. The ATR-FTIR spectrum further verified the presence of groups characteristic of fatty acids and triacylglycerols, with particular emphasis on the high degree of esterification of oil. For the first time, Langmuir monolayer studies were applied to JQSO, revealing its high compressibility, indicative of excellent emulsification properties. Furthermore, the viscoelastic modulus values suggested stronger intermolecular interactions, indicating that the oil monolayer is more resilient and capable of maintaining structural integrity under stress—an attribute often associated with oils rich in saturated or monounsaturated fatty acids. The presented data are in agreement with previously published results regarding the presence of antioxidants, including tocopherol and phenolic compounds. During the cold-pressing and subsequent analytical procedures used to obtain the oil, as well as during measurements, no organic chemicals such as hexane, chloroform, or methanol were used, nor was heat treatment applied; thus, this method may be considered a green chemistry approach. Finally, one general remark regarding the cold-pressed procedure is that this did not involve heat or chemical treatment; thus, all sensitive compounds prone to destruction or oxidation were preserved. Moreover, this production method supports sustainability by utilizing seeds that are often discarded during fruit processing, adding value to the agricultural chain and reducing waste.

## Figures and Tables

**Table 1:** DSC parameters (temperature and enthalpy) of crystallization and melting phase transition of Japanese quince oil and phase transition of Japanese quince seed cold-pressed oil.

	Onset Temperature (°C)			Peak Temperature (°C)	Total Enthalpy (J/g)		
Crystallization	Ton			T	ΔH		
	−50.1 ± 0.04			−52.9 ± 0.06	−35.3 ± 0.77		
Melting	Peak Temperature (°C)			Total enthalpy (J/g)	Partial enthalpy (J/g)		
	T1	T2	T3	ΔH	ΔH1	ΔH2	ΔH3
	−30.9 ± 0.04	−24.9 ± 0.05	−20.8 ± 0.01	55.9 ± 0.01	4.8 ± 0.06	28.0 ± 0.05	23.1 ± 0.00

T1–T3—peak temperature, ΔH1–ΔH3—enthalpy of three peaks indicated in Figure 1.

**Table 2:** The parameters designated from the isotherm profile: A_EXP_—extrapolated average surface area available to the molecule; A_C_—average surface area available to molecule at the collapse point; π_C_ surface pressure at the collapse point; C_S_^−1^—compression modulus; and ε_MAX_—surface viscoelasticity modulus.

Parameter	A_EXP_ [nm^2^]	A_C_ [nm^2^]	π_C_ [mN/m]	C_S_^−1^_MAX_ [mN/m]	ε_MAX_ [mN/m]
JQSO	1.49 ± 0.08	0.50 ± 0.03	36.2 ± 1.1	103.6 ± 1.9	100.9 ± 2.1

Explanatory notes: The data in the table are presented as the mean ± standard deviation (SD).

## Data Availability

The original contributions presented in the study are included in the article. Further inquiries can be directed to the corresponding authors.
